# A Sociodemographic variables questionnaire (Q-SV) for research on family caregivers of children with chronic disease

**DOI:** 10.1186/s40359-019-0350-8

**Published:** 2019-12-21

**Authors:** Filiberto Toledano-Toledano, Rocío Rodríguez-Rey, José Moral de la Rubia, David Luna

**Affiliations:** 1 Unidad de Investigación en Medicina Basada en Evidencias, Hospital Infantil de México Federico Gómez Instituto Nacional de Salud, Dr. Márquez 162, Doctores, Cuauhtémoc, 06720 México City, Mexico; 2 Universidad Europea de Madrid. Calle Tajo, s/n, 28670 Villaviciosa de Odón, Madrid, Spain; 3 Facultad de Psicología, Universidad Autónoma de Nuevo León. Dr. Carlos Canseco, 110, Esq. Dr. Aguirre Pequeño, Col. Mitras Centro, 64460 Monterrey, Mexico; 4 Comisión Nacional de Arbitraje Médico, Mitla No. 250-10° Piso, esq. Eje 5 Sur (Eugenia). Col. Narvarte, 03020, Benito Juárez, Mexico City, Mexico

**Keywords:** Social work, Expert judge, Social workers, Anxiety, Family caregivers, Pediatric chronic disease, Depression, Sociodemographic variables, Caregiver burden, Questionnaire, Quality of life

## Abstract

**Background:**

Chronic diseases in childhood can affect the physical and mental health of patients and their families. The literature on pediatric chronic diseases has found important associations between the sociodemographic variables of children and their caregivers and negative health consequences in families.

**Methods:**

In this study, we aimed to design and validate a questionnaire on sociodemographic variables that would be useful for research on pediatric chronic diseases; and investigate the relationship between sociodemographic variables and psychosocial variables among family caregivers. First, we created a questionnaire that consists of 20 demographic, medical, and family-related items based on a literature review and expert evaluations. This questionnaire was then validated by 335 expert reviewers in the field of Social Work, who work daily with the families of patients with chronic diseases in 10 National Institutes of Health of Mexico. The validation was based on three empirical criteria created specifically for this study, and the reviewers evaluated the usefulness, relevance, and permanence of the items. In a second cross-sectional, correlational and comparative study, a total of 446 family caregivers of children with chronic diseases were interviewed, and they completed the Sociodemographic Variables Questionnaire for research on family caregivers of children with chronic sociodemographic diseases and four psychosocial measurement instruments for evaluating anxiety, depression, caregiver burden and quality of life.

**Results:**

Based on the results of the first study, we created the Sociodemographic Variables Questionnaire (Q-SV) for research on family caregivers of children with chronic diseases, and it includes 17 items that assess demographic, medical, and family characteristics. The results of the second study showed that the 17 sociodemographic variables obtained in the validation by expert judges are useful for measuring and evaluating the relationship between psychosocial variables in families of children with chronic diseases.

**Conclusions:**

Psychosocial and sociodemographic factors are relevant for the development of research processes for families that care for children with chronic diseases.

## Background

Pediatric chronic disease represents one of the greatest challenges for family environments and leads to physical, psychological, socioeconomic, and behavioral effects on patients and their caregivers [1]. A chronic disease can be defined as a medical condition that continues or occurs again and again for a long time and may worsen over time [2]. The most common types of chronic disease in children are asthma, diabetes, obesity, migraine, epilepsy, developmental disabilities, and cancer [3]. In recent years, the prevalence in Mexico of pediatric chronic disease in children under 18 years of age has reportedly increased [4], which in turn has led to an increase in the number of family caregivers for this population.

In the research literature, a family caregiver is defined as a person who has a significant emotional bond with the patient; this caregiver is a family member who is a part of the patient’s family life cycle, offers emotional-expressive, instrumental, and tangible support, and provides assistance and comprehensive care during the chronic illness, acute illness, or disability of a child, adult, or elderly person [5].

In the case of children with chronic disease, the international literature indicates that the main family caregivers are the parents, who experience adversity, risk and vulnerability associated with the consequences of care during chronic illness [6]. Recent empirical findings have confirmed the complexity and consequences of care in families of children with chronic diseases, mainly in quality of life [7–9]; well-being [10–14]; depression in family caregivers [5, 15–17]; caregiver burden in the families of children with chronic conditions [6, 18–21] and anxiety [1, 22, 23]; and resilience to disease as a process of positive adaptation despite the loss of health, which involves the development of vitality and skills to overcome the negative effects of adversity, risks, and vulnerability caused by disease [24]. All these aspects are important variables for the measurement and evaluation of families that care for children with chronic disease and family processes for overcoming adversity [6]. However, the psychosocial consequences and the impact of care on the families of children with chronic diseases are related to contextual factors, such as the sociodemographic variables of families and children. The family and social contexts of these sociodemographic variables are very useful for researching, measuring and evaluating the lives of families providing care under complex chronic conditions.

In this regard, substantial theoretical, clinical and empirical evidence supports the relevance and representativeness of sociodemographic variables (SVs) in the health, human, and social sciences [25]. In particular, the literature on chronic disease has found important associations between the SV of children and their caregivers and negative health consequences in families [26]. Socioeconomic, cultural, environmental, and sociodemographic factors can thus interact with and determine an individual’s health status [27, 28]. Furthermore, the main empirical findings of studies on chronic pediatric disease suggest that the health and quality of life of caregiver families are related to SVs, medical variables, environmental factors, and healthcare system efficiency [9, 29–32].

According to empirical findings, two main perspectives are used to investigate the relationship between sociodemographic characteristics and their effects on families: a) one perspective focuses on the patient’s sociodemographic characteristics and b) the other focuses on the family and caregiver’s sociodemographic characteristics. The first perspective considers variables such as the patient’s age [33], type of diagnosis [34], and time since diagnosis [35, 36]. The second perspective examines the caregiver’s sex, caregiver’s age, time spent providing care to the patient [37–39], socioeconomic status [40], number of other dependent children in the home [41], caregiver’s occupation [42], caregiver’s level of education [43], and financial burden [44]. In addition, previous research has found that the negative effects of caregiving significantly decrease as family [45, 46], social, physical, financial, and health support increases [47, 48].

Despite the empirical evidence, it is still necessary and essential to recognize the use and importance of SVs when studying chronic diseases and their impacts on families. However, theoretical, clinical, or empirical consensus has not been reached regarding which SV should be included in research on individuals, groups, and families of children with chronic conditions [1, 9]. In Mexico and the international literature, measurement instruments that assess individual, family and sociocultural variables for research on families of children with chronic diseases have not yet been developed. Thus, this field of knowledge presents significant gaps that have important implications for developing research and intervention processes for families based on the real context in which families live and overcome adversity related to childhood disease.

Therefore, questionnaires on SVs that have been analyzed and evaluated by expert judges in the Social Work field according to the usefulness, relevance and permanence of SVs for families are lacking in the research literature, and such measurement instruments have not been used to study the complexity of the social context of family life and its relationship with the psychosocial variables of family caregivers of children with chronic disease. This existing gap in the literature of pediatric psychology can increase the difficulty of identifying risk factors associated with the pathological conditions to which the family caregiver is exposed and the consequences of care that such conditions imply.

The aim of this study was twofold: Study 1 was a qualitative study performed to design a questionnaire with relevant SVs for use in research with families of children with complex chronic diseases. The objective of Study 1 was to select the demographic, medical, and family variables that were the most relevant to include in the Q-SV in accordance with the scientific literature and the empirical criteria of expert reviewers in the field of Social Work belonging to 10 of the 13 National Institutes of Health of Mexico, where patients receive treatment and hospitalization for chronic disease. Study 2 sought to identify the relationship between the SVs measured by the Q-SV and four psychosocial variables: anxiety, depression, caregiver burden and quality of life. These psychosocial variables have been consistently reported in the literature as variables associated with the impact and consequences of care on families of children with chronic disease.

## Methods

### Ethical considerations

The Research, Ethics, and Biosafety Commission at the Hospital Infantil de México Federico Gómez National Institute of Health, Mexico City approved the protocol of the present study. This study was conducted in accordance with the ethical rules and considerations for research with humans currently in place in Mexico [49] as well as those outlined by the American Psychological Association [50]. Participation in this study was voluntary, and prior to their enrollment, the study participants were informed of their rights according to the Helsinki Declaration [51]. The expert reviewers and all family caregivers were informed of the objectives and scope of the investigation as well as their research rights. The expert reviewers in Social Work and the family caregivers who agreed to participate in the study provided a signed letter of consent. Consenting caregivers were given instructions and completed the questionnaires independently while at their child’s hospital, and the battery of tests were individually administered.

- Study 1: Content validation of the Sociodemographic Variables Questionnaire (Q SV-20 items) for research on family caregivers of children with chronic disease *by expert reviewers in Social Work.*

### Participants

Among 375 experts in Social Work who are affiliated with the 13 National Institutes of Health of Mexico, experts belonging to only 10 National Institutes of Health of Mexico where patients with chronic diseases are received for hospitalization and medical treatment were selected. In these 10 high specialty hospitals, only Social Workers who perform daily work with families and family caregivers of patients with complex chronic diagnoses were selected. Second, Social Workers were contacted personally at the institute where they worked or via email. Using a non-probabilistic sampling technique for convenience, a total of 335 Social Workers were recruited to be expert judges in the present study, which represented a participation rate of 89.3%. Of the 335 reviewers, 77.3% (*n* = 259) were women; 22.7% (*n* = 76) were men; 97.3% (*n* = 326) had a bachelor’s degree in Social Work; 1.8% (*n* = 6) had a master’s degree in social work; and 0.9% (n = 3) had received technical level studies in Social Work. The criteria for inclusion in study 1 were voluntary participation in the study, academic training in social work and performance of their social work functions via interviewing families and caregivers of patients with chronic disease. The exclusion criterion was the refusal of voluntary participation. In addition, incomplete responses of the evaluation instrument Q-SV were eliminated. Because the participation of the expert judges in Social Work was voluntary, the participants were not compensated for their collaboration in this study.

### Procedure

Once the review of the theoretical and empirical literature was completed, a questionnaire with 20 items pertaining to individual, family, and sociocultural characteristics was designed to define the sociodemographic profile of families of children with chronic disease. Next, Social Workers from the ten National Institutes of Health were convened and selected. Once included in the study, the participants received (in person or via email) a document (in Spanish) explaining the purpose and scope of the research as well as the Q-SV questionnaire. The judges’ evaluations were classified into three categories, which were generated a posteriori through a thematic content analysis: 1) Relevance: it is necessary, essential and fundamental to ask these items in the context of pediatric chronic disease, and the answers include “Never”, “Sometimes”, and “Always”; 2) Utility: it is beneficial to ask these items to investigate pediatric chronic disease in this institution, and the answers include “Nothing”, “Regular”, and “Much”; and 3) Permanence: these SVs should continue to be assessed over time in research with families experiencing chronic health conditions, and the answers include “Definitely not”, “Maybe”, and “Definitely yes”.

### Instruments

The first version of the Q-SV included 20 items grouped into four domains: 1) SVs of the caregiver: age, sex, marital status, years married, education, religion, city of origin of caregiver, number of children, occupation, and monthly household income; 2) SVs of the patient: age and sex; 3) medical variables of the patient: diagnosis, medical services, amount of time hospitalized, and time since diagnosis; and 4) family variables: parental role, family type, family life cycle, and support networks. The criterion of retention of a variable was to score a minimum of 60% at the positive end of the continuum of responses in the dimensions evaluated by the reviewing expert judges in Social Work.

### Results of study 1

The results of study 1 are shown in Table 1, and they include the results of the validation agreement rate of 20 items of the Q-SV by 335 expert reviewers in Social Work.

**Table 1:** Validation of the first version of the Q-SV 20 items by 335 expert reviewers in Social Work

Validation of the 20 items	Relevance (%)	Utility (%)	Permanence (%)	Final decision
Sociodemographic variables of the caregiver				
Sex	68	66	75	*
Age	81	76	74	*
Education	93	86	86	*
City of origin of caregiver	11	31	41	*Eliminated*
Religion	67	61	67	*
Marital status	65	77	80	*
Years married	19	34	46	*Eliminated*
Number of children	60	80	78	*
Occupation	83	93	96	*
Monthly household income	99	97	100	*
Sociodemographic variables of the children				
Age	93	67	63	*
Sex	67	83	83	*
Medical variables of the patient				
Diagnosis	89	98	97	*
Medical service	66	55	58	*Eliminated*
Amount of time hospitalized	78	70	70	*
Time since diagnosis	82	72	70	*
Family variables				
Family role of caregiver	87	86	95	*
Family type	82	71	69	*
Family life cycle	92	89	93	*
Support networks	100	97	99	*

^*^Items kept in the questionnaire

Based on an analysis of the answers of the expert judges in Social Work, 3 items were eliminated: years married, medical service, and city of origin of caregiver. Therefore, the following questions were eliminated from the original version with 20 items: 3. How many years have you been married or living together; 11. In what medical service is your patient hospitalized; and, 13. What is your city of origin? According to the evaluation of the expert judges, the item ‘years married’, which refers to the time spent living with or married to a partner, is not a relevant aspect or useful and necessary to keep in the study questionnaire. In addition, the Social Workers consider that the above questions related to medical services and the city of origin of the caregiver, which refers to the geographical location where the family caregiver habitually resides, are not useful or relevant for research in this context.

Therefore, the findings of study 1 confirm that in the research processes with families of children with chronic disease, four dimensions must be considered: SVs of the caregiver; SVs of the patient; medical variables of the patient; and family variables of the caregiver. These four dimensions are distributed in 17 items in the final version of the questionnaire Q-SV 17 Items. The main contribution of this study is the expert opinion of Social Workers about the relevant variables to carry out research processes with family caregivers of children with chronic diseases, because the expert reviewers had the academic training and the experience of working with families in contexts of significant adversity due to illness. The final version of the Q-SV (Q-SV 17 Items). *See* Additional file 1*.*

- Study 2: Transversal, correlational, and comparative study of the association between the Q-SV 17 items and four psychosocial variables: anxiety, depression, caregiver burden, and quality of life.

### Participants

A non-probabilistic sampling technique was used to invite 500 family caregivers of children with chronic disease. The final sample included 446 caregivers, indicating a response rate of 89.2%. The inclusion criteria were the ability to read and write, role as a family caregiver of a patient with a chronic pediatric disease, agreement to voluntary participation in the study, and signing of the informed consent form. The exclusion criteria were the inability to read or write and rejection of voluntary participation in the study. The criteria for elimination were incomplete or partial answers for the battery of measuring instruments. No compensation was provided to the family caregivers or pediatric patients for participation in this study.

### Measures

The following measurement instruments were applied.

Q-SV (Q-SV 17 items) for research on family caregivers of children with chronic disease and a battery of 4 instruments that evaluated the psychosocial variables anxiety, depression, caregiver burden, and quality of life (see Table 2).

**Table 2:** Instruments for evaluating psychosocial variables

Instrument name	Author	No items	Factors/Dimensions	Answer options	Reliability α
Anxiety Inventory	Beck et al. [52] Validated with a Mexican population [53]	21	1) Subjective 2) Neurophysiological 3) Autonomic 4) Panic	0 (Little or none) to 3 (Severe)	0.92
Beck Depression Inventory (BDI-II)	Beck et al. [54] Validated in family caregivers of children with chronic diseases by Toledano-Toledano and Contreras-Valdez [5]	21	1) Somatic-affective 2) Cognitive	0 (Nothing) to 3 (Much)	0.91
Zarit Caregiver Burden Interview (ZCBI)	Zarit, Reever and Bach-Peterson [55] Validated with a Mexican population [56].	22	1) Impact of care on the caregiver 2) Caregiver-patient interpersonal relationship 3) Self-efficacy	0 (Never) to 4 (Always)	0.89
Quality of Life Inventory (Whoqol-Bref)	WHOQOL Group [57] Validated with a Mexican population by González-Celis and Sánchez-Sosa [58]	26	1) Physical health 2) Psychological health 3) Social relationships 4) Environment	1 (Very unsatisfied) to 5 (Very satisfied)	0.89

### Procedure

The family caregivers were contacted by the research team in the hospitalization rooms of the Hospital Infantil de México Federico Gómez National Institute of Health, where their children received medical treatment. Team members then asked caregivers to participate in the study, explained the objectives of the study and clarified any doubts by the caregivers. The participants were informed that their participation was voluntary and that their answers would remain confidential and anonymous. Caregivers who agreed to participate were required to sign an informed consent form and subsequently answer the measurement instruments. All the instruments were applied individually, and the participants answered questions on their own in a single session. All information and instruments were delivered in Spanish.

### Data analysis

Descriptive analyses were conducted to assess the sociodemographic characteristics of the study sample. Pearson’s product-moment correlation coefficients (*r*) were calculated to examine the linear relationship between two quantitative variables. The means of quantitative variables were compared using Student’s t-tests, and the equality of variance between groups was tested using Levene’s test. If the latter test was significant, then Welch’s t-test was performed. The measure of central tendency in an ordinal variable was compared between two independent groups using the Mann-Whitney U test. Chi square (*χ2*) tests of goodness of fit were used to detect significant differences in the frequency of occurrence of a variable (discrete uniform distribution as the expected probability distribution). Chi square (χ2) tests of independence were calculated to examine the relationship between two categorical variables. The hypothesis tests were two-tailed, and the level of significance was set at 5%. Statistical calculations were performed with the SPSS v.24, IBM Inc., Chicago, USA, and Excel 2007.

## Results

### Sociodemographic variables of the caregiver

The sociodemographic characteristics of the 446 family caregivers and the comparison between the two sexes are presented in Table 3. In the sample, 82.3% of the participants (*n* = 367) were women between 13 and 63 years old (M = 31.77, SD = 8.66), and 17.7% (*n* = 79) were men between 18 and 56 years old (M = 34.22, SD = 8.64). Significantly more caregivers were female than male (χ2 = 185, df = 1, *p* < .01), and female caregivers were younger than male caregivers (t = − 2.27, df = 444, *p* < .05). The highest level of education was mainly secondary (44.2%, *n* = 197), followed by high school (25.8%, *n* = 115), and primary (18.8%, *n* = 84). Few participants had not received any schooling (3.4%, *n* = 15), and few had attended university (7.8%, *n* = 35). In addition, 92.4% (*n* = 412) followed a religion. Catholic Christian was the most frequent religion (80.9%, *n* = 361), followed by non-Catholic Christian (11.7%, *n* = 52). The remainder did not follow any religion. Among the caregivers, 77.5% (*n* = 346) were married or in a free union, 11.9% (*n* = 53) were divorced or separated, 7.6% (n = 34) were single mothers, and 1.3% (*n* = 6) were widowers; the remainder did not provide a response. Including the pediatric patient in their care, the participants had between 0 and 10 children (M = 2.32, SD = 1.23). The majority were dedicated to home care (65.5%, *n* = 292), while 26.5% (*n* = 118) reported some economically remunerated activity, with the remainder unemployed (7%, *n* = 31) or students (1.1%), *n* = 5). The average monthly family income was less than US $ 405.00 per month in 96.9% of participants (*n* = 432). The average number of children was two, with no difference between men and women (t = 0.70, df = 444, *p* = 0.483).

**Table 3:** Sociodemographic characteristics of the 446 family caregivers and differences between women and men

Variable		Women	Men	Total	Test statistic	*p*-value
Sex		367(82.3%)	79 (17.7%)	446 (100%)	χ2 = 185.97	< 0.001
Age	Years	31.77 ± 8.67	34.22 ± 8.64	32.21 ± 8.70	t = −2.27	0.024
Number of children		2.34 ± 1.24	2.23 ± 1.22	2.32 ± 1.23	t = 0.70	0.483
Educational level	None	14 (3.8%)	1 (1.3%)	15 (3.4%)	Z_U_ = -0.67	0.503
	Elementary	69 (18.8%)	15 (19%)	84 (18.8%)		
	Secondary	163(44.4%)	34 (43%)	197 (44.2%)		
	High school	92 (25.1%)	23 (29.1%)	115 (25.8%)		
	Graduate	27 (7.4%)	6 (7.6%)	33 (7.4%)		
	Postgraduate	2 (0.5%)	0 (0%)	2 (0.4%)		
Religion	Catholic Christian	293 (79.8%)	68 (86.1%)	361 (80.9%)	χ2 = 4.17	0.124
	Non − Catholic Christian	48 (13.1%)	4 (5.1%)	52 (11.7%)		
	None	26 (7.1%)	7 (8.9%)	33 (7.4%)		
Marital status	Married	143 (39%)	36 (45.6%)	179 (40.1%)	χ2 = 14.84	0.022
	Cohabiting	133 (36.2%)	34 (43%)	167 (37.4%)		
	Separated	36 (9.8%)	4 (5.1%)	40 (9%)		
	Single mother	34 (9.3%)	0 (0%)	34 (7.6%)		
	Divorced	11 (3.0%)	2 (2.5%)	13 (2.9%)		
	Widowed	6 (1.6%)	0 (0%)	6 (1.3%)		
	No answer	4 (1.1%)	3 (3.8%)	7 (1.6%)		
Occupation	Homemaker	291 (79.3%)	1 (1.3%)	292 (65.5%)	χ2 = 186.96	< 0.001 < 0.001^*^
	Office employee	34 (9.3%)	26 (32.9%)	60 (13.5%)		
	Trader	21 (5.7%)	22 (27.8%)	43 (9.6%)		
	Unemployment	13 (3.5%)	18 (22.8%)	31 (7%)		
	Worker	4 (1.1%)	11 (13.9%)	15 (3.4%)		
	Student	4 (1.1%)	1 (1.3%)	5 (1.1%)		
Average monthly family income (US dollars)	< 135	230(62.7%)	40 (50.6%)	270 (60.5%)	Z_U_ = -1.96	< 0.05
	[135, 270)	78 (21.3%)	22 (27.8%)	100 (22.4%)		
	[270, 405)	49 (13.4%)	13 (16.5%)	62 (13.9%)		
	[405, 540)	4 (1.1%)	3 (3.8%)	7 (1.6%)		
	[540, 810)	3 (0.8%)	1 (1.3%)	4 (0.9%)		
	≥ 810	3 (0.8%)	0 (0%)	3 (0.6%)		

*n* (%) = absolute frequency and percentage, M ± SD = arithmetic mean and standard deviation. Test statistic: χ2 = statistic value for Pearson’s Chi-squared test, t = statistic value for Student’s t-test, Z_U_ = z-statistic value for Mann-Whitney’s U-test, and p-value = probability value for a two-tailed test. * The exact probability was used because the requirement that at least 80% of the expected frequencies were greater than or equal to 5 was not met

### Sociodemographic variables of the children with chronic diseases

Among the children with chronic diseases attended by a family caregiver, the ages were between 1 and 17 years, 48% (*n* = 214) were girls with a mean age of 6.04 years (SD = 5.20), and 52% (*n* = 232) were boys with a mean age of 5.86 years (SD = 4.96). Significant differences between sexes were not observed in the frequency and mean age (Table 4).

**Table 4:** Sociodemographic characteristics of the 446 patients and differences between girls and boys

Variable	Girls	Boys	Total	Test statistic	*p*-value
Sex	214 (48%)	232 (52%)	446 (100%)	χ2 = 0.73	0.394
Age (years)	6.04 ± 5.20	5.86 ± 4.96	5.95 ± 5.07	t = 0.38	0.702

n (%) = absolute frequency and percentage, M ± SD = arithmetic mean and standard deviation. Test statistic: χ2 = statistic value for Pearson’s Chi-squared test, t = statistic value for Student’s t-test, and p-value = probability value for a two-tailed test

### Medical variables of the patients

Regarding clinical diagnosis, 74% (*n* = 330) of the 446 patients had an oncologic disease. Acute lymphocytic leukemia was the most frequent cancer. Moreover, 7% (*n* = 31) had abnormal blood flow due to a congenital heart defect, 4.7% (*n* = 21) nephrotic syndrome, 4% (*n* = 18) end-stage renal disease, 2.9% (*n* = 13) asthma, 2.7% (*n* = 12) tricuspid atresia, 2% (*n* = 9) Down syndrome, 0.9% (*n* = 4) tetralogy of Fallot, 0.7% (n = 3) HIV/AIDS, 0.7% (n = 3) organ transplant care, and 0.4% (n = 2) cystic fibrosis. There was no difference in diagnosis between the sexes.

It should be noted that Down syndrome is not a chronic disease. Nevertheless, cardiologists, endocrinologists, and other medical specialists refer children with Down syndrome to our service due to the complexity of the treatment of medical complications that this genetic syndrome entails. On the other hand, there were no cases of diabetes, which is a chronic disease; because these patients are treated in another service specialized in this endocrine disease.

Among the patients, 62.3% (*n* = 278) were hospitalized for up to a week, 30.1% (*n* = 134) from one week to six months, and 7.6% (*n* = 34) for greater time periods. The time elapsed since diagnosis was up to one year for 67.3% (*n* = 300) and longer for the remaining 32.7% (*n* = 146). There were no mean differences in time elapsed since diagnosis between the sexes, but the mean hospitalization time was longer for girls than for boys (Table 5).

**Table 5:** Clinical variables of the 446 patients and differences between girls and boys

Variable		Girls(n = 214)	Boys(*n* = 232)	Total	Test statistic	*p*- value
Diagnosis	Oncologic disease	171 (79.9%)	159 (68.5%)	330 (74%)	χ2 = 14.29	0.1600.145^*^
	CHD	14 (6.5%)	17 (7.3%)	31 (7%)		
	Nephrotic syndrome	6 (2.8%)	15 (6.5%)	21 (4.7%)		
	ESRD	8 (3.7%)	10 (4.3%)	18 (4%)		
	Asthma	6 (2.8%)	7 (3%)	13 (2.9%)		
	Tricuspid atresia	3 (1.4%)	9 (3.9%)	12 (2.7%)		
	Down syndrome	1 (0.5%)	8 (3.4%)	9 (2%)		
	Tetralogy of Fallot	1 (0.5%)	3 (1.3%)	4 (0.9%)		
	HIV/AIDS	2 (0.9%)	1 (0.4%)	3 (0.7%)		
	Organ transplant	1 (0.5%)	2 (0.9%)	3 (0.7%)		
	Cystic fibrosis	1 (0.5%)	1 (0.4%)	2 (0.4%)		
Hospitalization time	≤ 1 w	131 (61.2%)	147 (63.4%)	278 (62.3%)	Z_U_ = -0.98	0.328
	> 1 w & ≤ 1 m	43 (20.1%)	55 (23.7%)	98 (22%)		
	> 1 m & ≤ 6 m	16 (7.5%)	20 (8.6%)	36 (8.1%)		
	> 6 m & < 1 y	7 (3.3%)	2 (0.9%)	9 (2%)		
	≥ 1 y & < 2 y	3 (1.4%)	3 (1.3%)	6 (81.3%)		
	≥ 2 y	14 (6.5%)	5 (2.2%)	19 (84.3%)		
Diagnosis time	< 3 m	57 (26.6%)	56 (24.1%)	113 (25.3%)	Z_U_ = -0.406	0.685
	≥ 3 m & < 6 m	25 (11.7%)	23 (9.9%)	48 (10.8%)		
	≥ 6 m & < 1 y	26 (12.1%)	31 (13.4%)	57 (12.8%)		
	≥ 1 y & < 3 y	32 (15%)	50 (21.6%)	82 (18.4%)		
	≥ 3 y & < 5 y	36 (16.8%)	26 (11.2%)	62 (13.9%)		
	≥ 5 y & < 10 y	16 (7.5%)	22 (9.5%)	38 (8.5%)		
	≥ 10 y	22 (10.3%)	24 (10.3%)	46 (10.3%)		

n (%) = absolute frequency and percentage, M ± SD = arithmetic mean and standard deviation. Test statistic: χ2 = statistic value for Pearson’s Chi-squared test, Z_U_ = z-statistic value for Mann-Whitney’s U-test, and p-value = probability value for a two-tailed test. * The exact probability was used because the requirement that at least 80% of the expected frequencies were greater than or equal to 5 was not met. Diagnosis: ESRD = end-stage renal disease, CHD = congenital heart defect, and HIV/AIDS = human immunodeficiency virus/acquired immunodeficiency syndrome. Time: w = week, m = month, and y = year

### Family variables

The most frequent caregiver was the mother (77.1%, *n* = 344) or the father (16.1%, *n* = 72), and another type of relationship with the pediatric patient was observed in the remaining 6.7% of cases (*n* = 30). The family type of origin was nuclear in 50.4% of the cases (*n* = 225) and single-parent or of another type in 16.6% (*n* = 74) and 32.9% (*n* = 147), respectively. These families had small children (32.7%, *n* = 146) or children of school age or adolescents (59.2%, *n* = 264), and only 8% (*n* = 36) had adult children. Finally, their support networks were the family itself (83.2%, *n* = 371), assistance institutions (11.2%, *n* = 50) or other relationships in the remaining cases (5.6%, *n* = 25).

An analysis was performed of the variables obtained via the Q-SV 17 items and psychosocial variables. Table 6 shows the frequency and percentage of anxiety and depression levels among the caregivers. It should be noted that almost 90% of the caregivers had at most a moderate level of anxiety and depression.

**Table 6:** Levels of anxiety and depression in family caregivers

	Anxiety		Depression	
	n	%	n	%
Minimum	123	27.6	60	13.5
Mild	162	36.3	163	36.5
Moderate	105	23.5	157	35.2
Severe	56	12.6	48	10.8
No information	0	0	18	4

No significant mean differences (*p* > .05) were observed in the scores obtained for the scales of depression, anxiety and burden of the caregiver between the groups defined by the sex of the caregiver, the sex of the pediatric patient and marital status (married/free union versus other status). Similar findings were observed for quality of life and the perception of the state of general health, physical health, psychological health, social relations and environment, which are dimensions of the Whoqol-Bref instrument (p > .05). The once was the dimension related to social relations (t = 1.89, df = 444, *p* = .05), for which caregivers with a partner scored higher (M = 10.66, SD = 2.26) than those without a partner (M = 10.19, SD = 1.94).

The correlation between sociodemographic variables and psychosocial factors is shown in Table 7. The results show that only the correlation between the caregiver’s age and quality of life is significant. However, because the strength of their association is very low, this correlation may lack practical relevance. For levels of depression or anxiety and the level caregiver burden (high or low), an association was not observed (*p* > .05) with the sex of the caregiver and their role (parents vs. other), sex of the pediatric patient, family life cycle or type of family, disease diagnosis (cancer vs. other), time elapsed since diagnosis, religion, and income. Although no association was observed between the time elapsed since hospitalization and the level of anxiety (p > .05), a higher number of caregivers were diagnosed with severe depression when this time period was greater than one week (χ2 = 11.55, df = 3, *p* < .01).

**Table 7:** Correlation between sociodemographic and psychosocial variables

Variable	Anxiety	Depression	Caregiver burden	Quality of life
Sociodemographic variables of the caregiver				
Age	−.08	−.03	−.06	.10*
Number of children	−.07	.01	.03	−.20
Sociodemographic variables of the children				
Age	−.01	−.02	.03	.03

Pearson’s correlation product-moment coefficient (r). * Probability value less than .05 in a two-tailed test for the null hypothesis (H_0_): r = 0

## Discussion

The objectives of the present study were twofold: (a) Design a SV questionnaire (the Q-SV), and (b) investigate the relationship between these SVs and family caregivers’ psychosocial outcomes, including anxiety, depression, caregiver burden, and quality of life. The results of study 1 yielded the final 17-item version of the Q-SV. One of the benefits of this questionnaire is that the variables were evaluated by a large sample of Social Work experts, who have extensive contact and experience in dealing with family caregivers of patients with chronic diseases.

The results of study 2 provided further empirical support for the importance of examining the effect of SV on psychological outcomes among family caregivers of children with chronic diseases [5, 6, 24, 26, 46, 59–61]. The findings of study 2 are consistent with previous studies that reported a positive association between the duration of the child’s hospitalization and caregivers’ level of depression [5, 36, 43]. However, they are inconsistent with other studies in which a positive association was observed among age, economic status, and quality of life in caregivers [40, 62, 63]. It is important to note that compared with the other SVs measured in this study, the duration of child’s hospitalization had a greater negative effect on the caregivers’ depression levels.

Although a large set of associations or insignificant differences were detected in this study, we must consider that psychopathological variables, such as depression, anxiety and caregiver burden, were evaluated, and evidence indicates that under unfavorable conditions, caregivers of pediatric patients are able to develop a resilience that allows them to reduce or avoid psychopathological symptoms and thus be functional in the circumstances that are presented [1, 24, 61]. Thus, it is possible that the caregivers in this study also developed a degree of resilience that served as a protective factor for psychopathological conditions. Future studies should explore variables that are more related to well-being and optimal functioning (e.g., psychological well-being). Additionally, although certain results were not significant, they demonstrate that the Q-SV variables can be used to perform statistical analyses with psychosocial variables. Such analyses can be used to detect the effect of SVs and the absence of effects of psychosocial variables. Future studies should investigate the usefulness of the variables included in the Q-SV and eliminate those that are not relevant and incorporate others that are not currently included.

In conclusion, the Q-SV is considered to include the most representative and essential sociodemographic characteristics for studying the impact of pediatric chronic illness on family caregivers. In addition, the Q-SV can help determine which SV to include in research on families of children with chronic disease, and it allows researchers to collect data on important individual, family, and sociocultural factors that characterize families of children with chronic disease. However, the selection of SVs in each study should be determined by the specific study objectives. Finally, the present study sought to develop an empirically supported, useful, and valid questionnaire on SVs that could be used in the context of chronic pediatric health conditions. The Q-SV can be used by researchers to collect relevant demographic data among participants. Furthermore, the Q-SV can be used to detect areas of intervention for caregivers of children with chronic diseases because family members of children with chronic diseases are subject to stress, adversity and risk and vulnerable to negative health outcomes. Finally, for future research with families, the number of hours that family caregivers devote per day to caring for their patients should be included because this is an important SV that may be related to the psychosocial variables that influence the caregiver’s burden and the psychosocial effects of caring for children with chronic disease.

### Limitations

The present study should be considered within the context of its limitations. First, the sample size was small and not representative of the overall population. Second, the use of non-probabilistic sampling limits the external validity and generalizability of the results. Third, the majority of pediatric patients included had some type of cancer; this may be useful to develop future research on family caregivers of children with cancer. Despite these limitations, the findings of the present study indicate that contextual factors must be considered when assessing the well-being of family caregivers.

## Conclusions

Future studies might apply the Q-SV to other samples of diverse populations from both the public and private sectors and beyond the social services of the National Institute of Health. In addition, future researchers may implement this useful, valid and culturally relevant tool for conducting research on pediatric chronic diseases and their effects on families. The Q-SV could be used as a screening tool by social workers to collect SVs of the families of children with chronic conditions and their family caregivers. Furthermore, this tool could be used to identify the effects of the Q-SV variables on patient anxiety and depression as well as caregiver burden and quality of life.

## Supplementary information

**Additional file 1.** A Sociodemographic Variables Questionnaire (Q-SV) for Research on Family Caregivers of Children with Chronic Disease.

## Data Availability

The datasets used and analysed during the current study available from the corresponding author on reasonable request.

## Abbreviations

Q-SV: Sociodemographic Variables Questionnaire
USD: United States Dollars
