# Supplementary material for: A Sociodemographic variables questionnaire (Q-SV) for research on family caregivers of children with chronic disease
Source: BMC Psychol. 2019 Dec 21;7:85. doi: 10.1186/s40359-019-0350-8 (PMC6925508; doi:10.1186/s40359-019-0350-8)
Supplement: Supplementary file 1 — Additional file 1. A Sociodemographic Variables Questionnaire (Q-SV) for Research on Family Caregivers of Children with Chronic Disease. [file 40359_2019_350_MOESM1_ESM.docx]

**Annex.**

**A Sociodemographic Variables Questionnaire (Q-SV) for Research on Family Caregivers of Children with Chronic Disease**

Final version: 17- items

| 1. What is your age? |
| --- |
| 2. Sex: |
| ☐ Male |
| ☐ Female |
| 3. What is the highest level of education you have completed? |
| ☐ Never attended school |
| ☐ Primary education |
| ☐ Secondary education |
| ☐ Technical |
| ☐ Preparatory |
| ☐ College/Bachelors |
| ☐ Postgrad |
| 4. What is your religion? |
| ☐ Catholic Christian |
| ☐ Non-Catholic Christian |
| ☐ None |
| ☐ Other |
| 5. How many children do you have? |
| 6. What is your occupation? |
| ☐ Homemaker |
| ☐ Worker |
| ☐ Merchant |
| ☐ Employed |
| ☐ Student |
| ☐ Unemployed |
| 7. What is the diagnosis of your patient? |
| 8. How long has the patient been hospitalized? |
| 9. Patient sex: |
| ☐ Female |
| ☐ Male |
| 10. Patient age: |
| 11. Marital status: |
| ☐ Married |
| ☐ Living together/Co-habitation |
| ☐ Separated |
| ☐ Divorced |
| ☐ Single mother/father |
| ☐ Widow/Widower |
| ☐ Other |
| 12. What is your relationship to the patient? |
| ☐ Mother |
| ☐ Father |
| ☐ Sibling |
| ☐ Aunt/Uncle |
| ☐ Grandparent |
| 13. Who lives at home? |
| ☐ Mom, dad, and children |
| ☐ Parents, children, and grandparents |
| ☐ Parents, children, grandparents, aunts/uncles, and cousins |
| ☐ Father only and children |
| ☐ Mother only and children |
| ☐ With other families |
| 14. Indicate the composition of your family at home? |
| ☐ Family with small children |
| ☐ Family with school-age children |
| ☐ Family with adult children |
| 15. How long ago was your patient diagnosed? |
| ☐ A few weeks ago |
| ☐ 3 months ago |
| ☐ 6 months ago |
| ☐ 1 year ago |
| ☐ 3 years ago |
| ☐ 5 years ago |
| ☐ 10 years ago |
| 16. From where do you primarily receive support? |
| ☐ Family |
| ☐ Friends |
| ☐ Business |
| ☐ Religion |
| ☐ Government |
| ☐ Non-governmental organizations |
| 17. What is the household’s monthly income? |
| ☐ Between USD 120 and USD 160 |
| ☐ Between USD 161 and USD 350 |
| ☐ Between USD 351 and USD 520 |
| ☐ Between USD 521 and USD 800 |
| ☐ More than USD 1000 |
